# Production and verification of the first Atlantic salmon (*Salmo salar* L.) clonal lines

**DOI:** 10.1186/s12863-020-00878-8

**Published:** 2020-07-08

**Authors:** Tom Johnny Hansen, David Penman, Kevin Alan Glover, Thomas William Kenneth Fraser, Tone Vågseth, Anders Thorsen, Anne Grete Eide Sørvik, Per Gunnar Fjelldal

**Affiliations:** 1 Institute of Marine Research, 5984 Matredal, Norway; 2 Institute of Aquaculture, School of Natural Sciences, University of Stirling, Stirling, FK9 4LA Scotland, UK; 3 Institute of Marine Research, 5817 Bergen, Norway; 4 Department of Biology, University of Bergen, Bergen, Norway

**Keywords:** Meiotic and mitotic gynogenesis, Doubled haploids, Clones, Microsatellite, Fish

## Abstract

**Background:**

In several fish species homozygous and heterozygous clonal lines have been produced using gynogenetic and androgenetic techniques. These lines are standardized and can be reproduced over generations. In rainbow trout such lines have existed for decades and has become important research tools in genome studies as well as in studies of commercially important traits. The Atlantic salmon is one of the best studied fish species globally, but all experiments are done on fish of wild or domesticated origin and access to standardized immortal fish lines would be of great benefit. Here, we describe the protocols developed to produce mitotic gynogenes, and from these the first clonal lines in Atlantic salmon.

**Results:**

Atlantic salmon eggs fertilized with UV irradiated sperm combined with a pressure shock applied at 4700–4800 minC at 8 °C gave all homozygous (doubled haploid) gynogenetic progeny with high survival. From the six first maturing females, five all homozygous clonal lines were produced by meiotic gynogenesis and were verified as clonal and identical to their mother with microsatellite markers.

**Conclusions:**

We have now produced the first documented cloned Atlantic salmon lines. This work demonstrates the potential for production of further Atlantic salmon clonal lines, potentially with distinct characteristics. Such lines will provide an important resource for further elucidation of phenotypic and genetic traits in this globally important species.

## Background

In fish, progeny after parthenogeneic development can be viable [1] and after the pioneering work on zebrafish, *Brachydanio rerio* [2], protocols for controlled production of meiotic gynogenetics and double haploid mitotic gynogenetics and androgenetics have been developed (reviewed by [1, 3]). In several species of fish (e.g. zebrafish [2]; Nile tilapia, *Oreochromis niloticus L*. [4]; Common carp, *Cyprinus carpio* [5]; amago salmon, *Oncorhynchus rodurus* [6]; rainbow trout, *Oncorhynchus mykiss* [7, 8], homozygous or heterozygous clonal lines have also been produced from the double haploid mitotic gynogenetics and/or androgenetics. These doubled haploids and/or clones have multiple applications as research animals, as they are standardized and can be reproduced over generations (reviewed by [3, 9]).

Among the salmonids, both rainbow trout (*Oncorhynchus mykiss*) and Atlantic salmon (*Salmo salar*) have become important aquaculture production animals. In rainbow trout, doubled haploid and clonal lines were established early (see [7, 8]) and have been utilized in the production of linkage maps [10, 11], detection of QTLs for meristic traits [12] and investigations of developmental rate [13]. They have also proved valuable in detailed analysis of commercially important traits such as disease resistance and utilization of animal contra vegetable dietary sources. Different rainbow trout clones show a wide range of susceptibility to viruses like the infectious salmon anaemia virus [14], the rhabdoviruses viral haemorrhagic septicaemia virus (VHSV) and infectious haematopoietic necrosis virus (IHNV) [15], and have been used to identify a major QTL for resistance to VHSV [16]. They also show a wide range of susceptibility to the bacteria *Flavobacterium psychrophilum* and have been used to gain insights into the genetic basis of the rainbow trout’s natural resistance to this bacteria [17]. In a pioneering study on utilization of marine/vegetable dietary sources [18], clonal rainbow trout lines were used to describe a genotype x protein source interaction in feed intake and feed efficiency and final weight following two feeding periods. A follow-up study describe how an early short-term exposure of fry improved the acceptance and utilisation of the same diet at a later life stage [19].

Atlantic salmon is also one of the most domesticated fishes [20, 21], and is also one of the best studied globally with extensive genomic resources [22]. A wide variety of experiments have been conducted on this species, using fish of wild and domesticated origin. However, for a long time it has been noted that access to standardized and genetically defined fish would greatly benefit the scientific community [23]. Production of meiotic gynogenetics using ^60^Co irradiated sperm and cold shock was unsuccessful [24], but the use of ^60^Co irradiated Atlantic salmon sperm [25] and UV light (UV) irradiated rainbow trout sperm [26], combined with heat shock, produced meiotic gynogenetics. Later, mitotic gynogenetics were produced [27] using UV irradiated milt and pressure shocks, but the progeny was terminated after 24 months and the production of clonal lines was not attempted.

In the present study we describe the development of a protocol for production of Atlantic salmon mitotic gynogenetic fish, and compare their growth with outbred progeny from the same broodfish and describe their phenotypic development and sexual maturation. We also present data from the production of isogenic lines from the first females that entered sexual maturation and the genotypic and phenotypic evaluation of these lines.

## Results

### Optimizing the mitotic gynogenesis protocol

In the eggs that were fertilized with unirradiated milt to determine the first cleavage interval (FCI), the first signs of cleavage were found after 750 mins (6000 minC) and all eggs were at the 2 cell stage after 790 mins (6320 minC). Fifty % cleavage was between 6160 and 6240 minC and was set to 6200 minC.

In the two experiments attempting to optimize the mitotic gynogenesis protocol (Exp2011 and Exp2012), surviving progeny were found in all pressure treatments between 4400 and 5100 minC, with the highest survival at 4700 (Exp2012) and 4800 minC (Exp2011) at 8 °C, with only minor effects of UV protocol (Table 1). Mean survival until first feeding in the 4800 minC groups in Exp2011 (mean of 6 and 8 mins UV protocols) was 20.2% (or 23.3% of the survival in the controls). In Exp2012 the highest survival until first feeding was found at 4700 minC with 10.2% (12.3% of controls), but acceptable survival was also found at 4800 minC with 6.5% (7.8% of controls). However, early mortalities (mainly during first feeding) were considerable. In Exp2011, survival until tagging and DNA sampling (7 August 2012; 107 days after first feeding; mean weight 17.8 g) was 14.3% (16.7% of controls; mean of 2 UV protocols), and in Exp2012, survival until tagging and DNA sampling (18 September 2013; 180 days after first feeding; mean weight 41.2 g) was 7.9% for 4700 minC (9.6% of controls) and 5.7% for 4800 minC (6.9% of controls). After tagging mortality was very low and the reduction in numbers of fish (Additional file 1: Table S1) are mainly due to sampling and removal of stunted and deformed fish.

**Table 1:** Survival from fertilisation to first feeding/tagging (dates are given in the text) of groups of salmon eggs fertilised with UV light irradiated and unirradiated control sperm

Date	UV (mins)			Pressure induction time (minutedegrees postfertilisation)						
		< 4400	4400	4600	4700	4800	4900	5000	5100	Contr
14 Dec 2011	6	0/0	0.1/0.0	1.7/1.2		19.3/13.5				86.6/85.8
	8		0.3/0.2	2.8/1.7		21.0/15.1				86.6/85.8
12 Dec 2012	6				10.2/7.9	6.5/5.7	1.1/1.1	1.0/0.9	2.11/1.55	83.1/82.1

The female egg donor in Exp2011 was heterozygous for 15 of the 18 analyzed microsatellites (Additional file 2: Table S2) and the sperm donor had at least one allele that was not shared with the female for 16 of the 18 analyzed microsatellites. For eight of the microsatellites, the male and female did not share alleles. Microsatellite analysis from the August 2012 sampling (Additional file 2: Table S2) showed that the control group of offspring was heterozygous with markers from both male and female. Of the 319 fish from the treatment groups, 317 were homozygous for all markers and carried only alleles from the egg donor. Two individuals were heterozygous and also displayed alleles from the sperm donor. These two individuals, one of each sex, were removed from the experimental population. The control group had 23 females and 22 males.

**Fig. 1:**
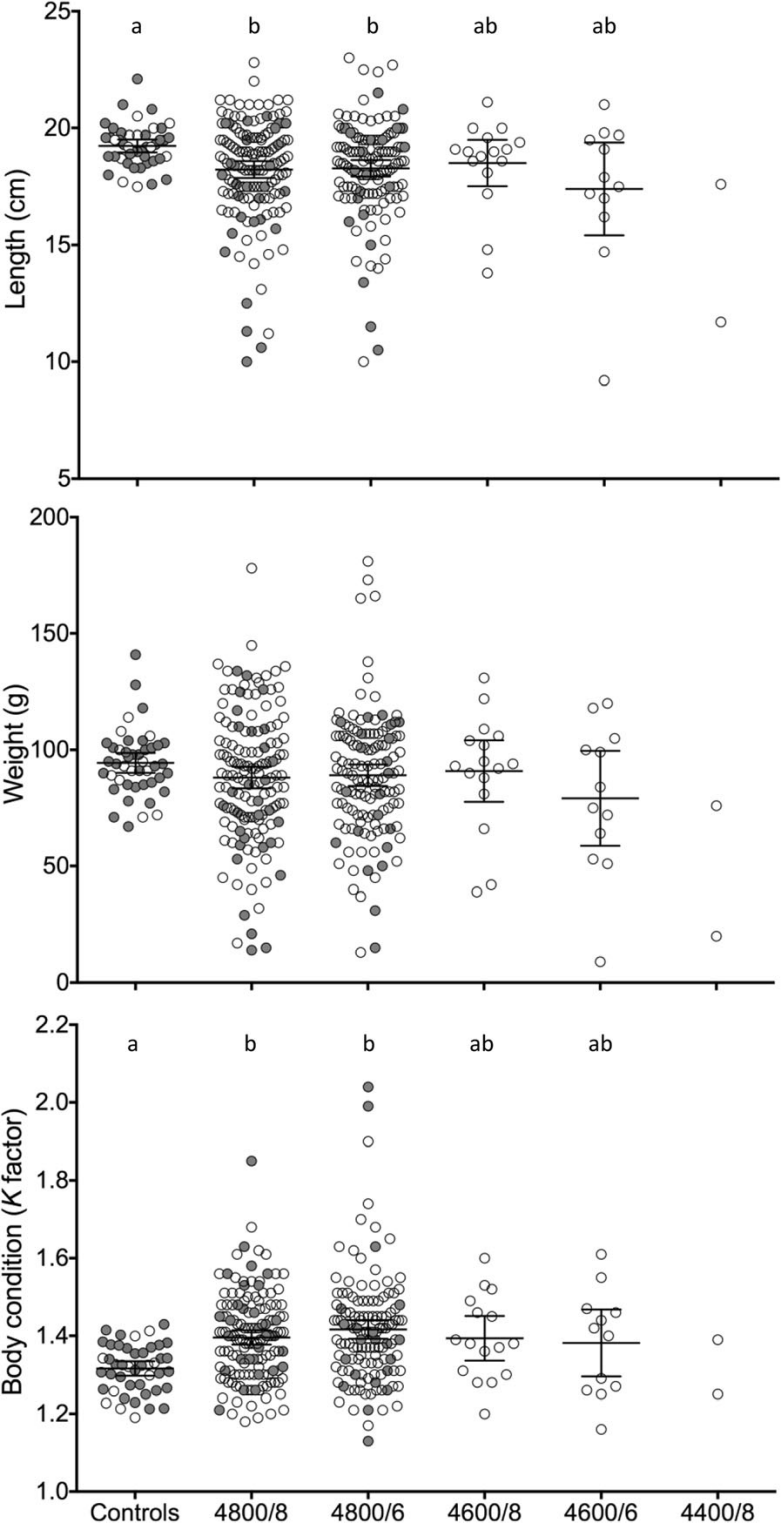
Mean ± 95% confidence interval of length, weight and condition factor of all experimental fish in Exp2011 on 28 November 2012 (day 220 of feeding). Gray circles are individuals that were euthanized on 28 November and open circles are individuals that were kept on until June 2013

The length, weight, condition factor, fish numbers and the sexual maturation for the fish from the controls and the different treatment protocols in Exp 2011 are presented in Additional file 1: Table S1. No significant differences in mean weight were found at any time either between the control group and the treatment groups (two first samples) or between the treatment groups at any time. The treatment groups had a much higher variation than the controls (cv 2–3 times higher; data not shown), with a high frequency of small fish (e.g. 20.3% of the treatment groups were smaller than the smallest fish in the control group in November 2012). However, some individuals also grew well with 5 individuals from the treatment groups being heavier than any of the controls (Fig. 1b). In November 2012 when the deformed fish and most of the controls were euthanized, the treatment groups were significantly shorter and had a higher condition factor than the controls (Fig. 1, Additional file 1: Table S1). The different treatment groups also had higher variation than the controls for all measured parameters (Additional file 1: Table S1; Fig. 1). After the deformed fish and most of the controls were euthanized (Fig. 1, Additional file 1: Table S1) no significant differences were found between the remaining groups at any time. The radiographs from June 2013 revealed vertebral deformities in 4 of the 30 individuals from the treatment groups with no deformities being found in the control fish. The deformities affected 2 to 4 vertebrae and were compressions, or combinations of compressions and fusions.

In 2014, six mature fish were found in the 4800/8 group and individuals matured also in 2015 and 2016, and a few fish were still immature when the study was terminated in 2016.

### Production of clonal lines

The six females that matured in 2014 (Table 2) gave between 4886 and 6706 eggs corresponding to between 1517 and 2261 eggs per kg bodyweight. Five of these lines gave surviving progeny and after 60 days of feeding survival varied between 3.9 and 28.3%. The two most abundant lines had 1795 (line 3) and 1764 (line 6) surviving progeny after 60 days of feeding. On 30 November (235 days of feeding), the mean weight of the lines varied between 49 g (line 5) and 125 g (line 6) and all individuals in all lines were females. Two of the lines (lines 5 and 6) had a very high incidence of vertebral deformities with ‘short tail’ being the most common (34.2% in line 5 and 29.7% in line 6). The high incidence of deformities was also reflected in the high K in line 5 and 6. Line 5 also had a high incidence of curvatures (36.8%) and 76.3% missed the pelvic fin. Line 3 had no deformities, good growth and the highest survival. Microsatellite analysis of the fish sampled on 16 November 2015 confirmed that all progeny were homozygous and identical to their mothers. This is with the exception of individuals 5 and 6 in cloned line 2 (Additional file 2: Table S2). For these two fish, one of the eighteen markers presented a single homozygoes allele that was 8 bp shorter than the mother’s single allele for that marker. The underlying cause(s) of this anomaly are not known. However, microsatellites are known to mutate through a slippage of one or two repeats in the motiv, in this case by 4-8 bp, and could explain the observation [28, 29].

**Table 2:** Length, weight and egg production of the six mitotic gynogenetic clonal line progenitors maturing in 2014 and the survival, growth and phenotypic description of their offspring. *Female 5 was only partly ovulated

	Line nr					
	1	2	3	4	5	6
Length (cm) of female	63.9	65.8	60.7	66.7	61.3	68.0
Weight (g) of stripped female	2790	2509	2803	3460	3220	4175
Eggs (N)	5469	4867	6340	6204	4886*	6706
Survival eyed stage (%)	73.6	39.5	72.8	0	44.0	97.9
Survival to first feeding (%)	36.2	24.6	43.5	0	8.2	65.7
Survival after 60 days feeding (%)	15.8	14.0	28.3	0	3.9	26.3
Survival after 60 days feeding (N)	864	680	1795	0	189	1764
Sampling 30 November 2015						
N sampled	28	24	30		38	37
Weight (g) (SD)	54 (14)	125 (30)	90 (25)		49 (28)	79 (25)
Condition factor K (SD)	1.18 (0.08)	1.24 (0.10)	1.24 (0.08)		1.39 (0.27)	1.47 (0.36)
Sex F/M	28/0	24/0	30/0		38/0	37/0
Missing pelvic	4				29	3
Short trunk	1	1			2	3
Short tail		1			13	11
Upper jaw					1	
Lower jaw			1			
Scoliosis					6	
Kyphosis					2	
Lordosis					6	3
Opercula						1

## Discussion

This study describes the methods and production of the first cloned Atlantic salmon lines. In two experiments fertilization with UV irradiated sperm combined with a pressure shock applied at 4700–4800 minC at 8 °C gave all homozygous (doubled haploid) gynogenetic Atlantic salmon, with survival rates ranging from 9.6 to 16.7% after 107 to 180 days of feeding. Based on the absence of paternal inheritance found in the microsatellite loci and the female sex of the progeny (Additional file 1), we conclude that both UV protocols (6 and 8 mins) used in Exp2011 gave a complete inactivation of the sperm DNA. Also, the homozygosity for all investigated loci in the progeny show that they are produced by first cleavage block gynogenesis and not spontaneous polar body retention. A blockage of the second meiotic division would leave some level of heterozygosity [30], as the female progenitor had a high level of allelic heterozygosity (15 out of 18 of the investigated loci).

The timing of the pressure shock in Exp2011 (3800 to 4800) were chosen after [27] who produced mitotic gynogenetics when activating salmon eggs with UV irradiated sperm and using pressure shock at 400 to 470 mins after fertilization at 10 °C, finding an optimized timing at 440 mins (4400 minC). In our two experiments we found an optimized induction time around 4700 to 4800 minC, corresponding to between 75.8 and 77.4% of the FCI (using the 6200 FCI found in Exp2011 which agrees well with the FCI of 6240 described earlier [31].

Optimal timing of a temperature or pressure shock has earlier been shown to coincide with the metaphase [2, 5, 32, 33] and/or prometaphase [34] of the mitosis, preventing the partitioning of the duplicated chromosomes into two cells. The result is a cell with two identical sets of chromosomes [2]: doubled haploids when using UV irradiated sperm, or tetraploids when using intact sperm. Our optimized timing agrees well with studies done on several other species, reported to be 65 ± 5% of the FCI in rainbow trout [35], 70–72.5% in brook trout [36], 70–75% in common carp [5], but differs from 45.8–57.2% in pressure-shocked Nile tilapia [37, 38]. However, variation between studies and species exist as FCI is dependent on temperature [39], increases during the spawning season and with postovulatory ageing of the eggs [35], can vary between populations [39, 40], from year to year in the same population [36] and varies between females [34].

The survival until first feeding of doubled haploids was considerably higher in Exp2011 (22–24% of controls) than in Exp2012 (12.3% of controls), however, both were in the range of the average of 19% found in rainbow trout [41] and 14–16% surviving swim-up fry in brown trout [42]. In studies on other species, survival varies considerably. Twentynine % normal-appearing embryos were found 24 h after fertilization in zebrafish [2], compared with 0–6% survival until hatching in Medaka [43], 3.5–15% normal fry 96 h after fertilization in common carp [5], < 1–26% feeding fry varying between females in loach (*Misgurnus anguillicaudatus*) [44], 6.9% of control at yolk sac resorption in Nile tilapia [4] and 12.8% until hatching in red sea bream [45]. However, these studies do not represent long-time survival as doubled haploid progeny often suffer high mortalities during early life. In [41] the progeny of the six best females of 15 had a mean survival until first feeding of approximately 30% which was reduced by approximately one third to 20% during the first 150 days of feeding, comparing well with the mortality of approximately 30% during the first 107 days of feeding in the present study (Table 1). Still this survival is high compared to other species, with 5.8% survival until adults in zebrafish [2], 0.2% in medaka [43], mortalities between 36.1 and 57.3% between hatching and 28 days post-hatching in common carp [5], mortality of 91.4% at 90 days in red sea bream [45] and survival of 155 of 323 hatching doubled haploid progeny in Nile tilapia [46]. The survival of doubled haploid progeny from 15 rainbow trout females varied between 0 and 53%, and it was hypothesized that genetic factors could explain part of the variation [41]. Generally, the main factors influencing on the yield/survival of doubled haploids are species-specific gene dosage compensation mechanisms, expression of early embryonic recessive homozygous deleterious mutations, egg quality and the occurrence of spontaneous absorption of the polar body creating heterozygous meiotic diploids [see reviews 3, 9]. In the present study the egg quality was good (high survival of controls) and no heterozygous meiotic diploids were observed, giving support to the hypothesis that genetic factors, i.e. occurrence of recessive deleterious alleles, was the main reason for the reduction in early survival.

In the present study, no significant differences in mean weight were found at any time between the control group and the treatment groups or among the treatment groups. However, our groups are based on one female progenitor. In rainbow trout, the mean weight of groups of mitotic gynogenetics were less than 80% of that of the diploid controls after 103 days of feeding [41], with the reduction varying between 4.1 and 30.9% dependent on the female progenitor. Also, the much higher variation than in the controls, the high frequency of small fish, the significantly higher condition factor, and the higher incidence of individuals with curved or shortened vertebral column in the doubled haploids agrees well with earlier studies. Red sea bream mitotic gynogenetics were compared with diploid controls for almost 3 years following hatching and the gynogentics had lower weight from year one on, a higher body depth, a much higher variance in measured parameters, and a higher incidence of short vertebral columns, scoliosis and deformities in the head [45]. In carp mitotic gynogenetics had lower weight and higher variance compared to controls [47] and doubled haploid tilapia had high incidence of deformities and retarded growth [46]. Our radiological examination in June 2013 revealed that 4 out of the 30 radiographed dh individuals had vertebral deformities which can affect the condition factor (see [48]). However, the number of affected fish and the low number of affected vertebrae is too low to explain the observed differences in condition factor between the doubled haploids and controls. The low incidence of deformities and also the fact that some of the doubled haploid individuals are heavier than the controls in November 2012 (Fig. 1b), indicate a potential for production of well-performing doubled haploids with a normal phenotype.

### Production of clonal lines

In this study five all homozygous clonal lines were produced by meiotic gynogenesis of eggs from doubled haploid progenitors and verified as clonal and identical to their mother with microsatellite markers.

Poor reproductive performance of doubled haploid progenitors has been highlighted as a major limitation in the production of isogenic lines [9, 44]. The relative fecundity (eggs/kg female bodyweight) of doubled haploid rainbow trout was normal and even significantly higher than controls [7], but with observations of sterile and hypofertile fish, and homozygous carp females had severe gonadal defects and less than 10% could be reproduced [5]. In our study, egg production of the female progenitors was high and well within what is normally seen in outbred Atlantic salmon (e.g. [49, 50]). However, early mortality was high, in accordance with studies on other species; e.g. between 3.3 and 36.9% of controls in tilapia [51], less than 5% survival until yolk sac resorption in Nile tilapia [52], between 0 and 70% survival until hatch in medaka [43], and 29 and 50% survival until first feeding at first and second spawning in rainbow trout [7].

The treatment protocol that is used, i.e. the UV-irradiation protocol of the sperm which can leave fragments of chromosomes [42, 53] and the pressure treatment of the eggs [3] can potentially contribute to the mortality in the clonal lines as well as in the production of the dh progenitors. However, both the UV-irradiation protocol and the pressure treatment protocol that was used in the present study are strictly standardized and cannot explain the variation in survival between the lines. Moreover, the pressure treatment protocol is used extensively in production of triploids both for aquaculture production and for research purposes. Different triploid induction protocols have been tested in brown trout [54] and Arctic charr (*Salvelinus alpinus*) [55], and despite a high variation in the parameters (pressure, timing and duration of pressure) they found a high triploidisation rate, and a survival that is high, and only occasionally significantly different from controls. If the pressure shock itself had some detrimental effect on egg/larval development and survival, one would expect this to be consistent over studies and in the case of our study give the same effect in all lines. Neither should the early mortality in production of homozygous clonal lines be influenced by lethal alleles because they have been eliminated in the first generation [9]. However, each line still represents only one haplotype (extreme inbreeding) and can contain homozygous alleles that are detrimental which again can be mirrored in the variation in phenotype and survival within and between lines as seen in the present material. Also, in our study all the dh females were stripped for eggs on the same day and the time of ovulation was not recorded. Hence, as post-ovulatory aging is an important determinant for egg quality (e.g. [56, 57] this could be an important variable leading to differences in survival. The importance of egg quality has also been demonstrated in rainbow trout where survival until first feeding increased from 29% in first time spawning doubled haploids to 50% in their second spawning [7]. Also, both the extreme inbreeding and possibly also a post-ovulatory aging of the eggs are factors that can contribute to the different morphological deviations and deformities that were seen in our clonal lines. The effect of inbreeding on body deformities is well described in studies on both salmon [58] and rainbow trout [59] and in fully homozygous fish the incidence can be considerable [53]. However, in salmon, deformations linked to post-ovulatory aging were mainly found in the head [57] and these were not seen in the present study.

## Conclusions

We have now produced the first documented cloned Atlantic salmon lines, and demonstrated the potential for production of further Atlantic salmon clonal lines, potentially with distinct characteristics. After thorough testing and description, a selection of these lines could make up an important resource of standardized animals in experimental studies and provide an important resource for further elucidation of phenotypic and genetic traits in this globally important species. At the moment we are keeping 11 lines at the institute, and they all originate from Exp2011 and Exp2012 described above. Line 3 which is described in this study has been reproduced in 2018 and 2019, and four lines that originated from Exp2012 were reproduced again in 2019. The lines are now a very important part of our research infrastructure.

## Methods

### Sperm irradiation protocol

The sperm irradiation protocol was developed in 2011 from the protocol for cod sperm [60], using the same equipment, the recommended dilution (1:40) and the same germicidal UV lamp (254 nm, 15 W, 220 V, 50 Hz). Milt was diluted with milt fluid (milt from several males were centrifuged until clear and the clear milt fluid was frozen and stored at − 20 °C and thawed before the experiment). 15 mL aliquots of the diluted milt was placed in a 9 cm petridish surrounded by ice and placed on top of a magnetic stirrer and irradiated at 0.48 mWcm^− 2^ (VLX-3.W radiometer, Cole Parmer, USA). The source-filter to sample distance was maintained at 20 cm throughout the experiments. Optimal irradiation dose (50% activity compared to diluted unirradiated sperm (see [60]), was found to be 6–8 min and cold freshwater was used to activate the sperm.

### Optimising the mitotic gynogenesis protocol

All experiments were done with eggs from the domesticated and commercially available Aquagen strain, Aqua Gen AS, Trondheim, Norway. On 14 December 2011 (Exp2011), 4 ml of milt from one male salmon was diluted with 160 ml milt fluid (1:40). Twelve 15 ml aliquots of the diluted milt were irradiated with UV light for 6 or 8 mins in a 9 cm petridish at 0.48 mWcm^− 2^ and transferred to 25 ml polyethylene (PE) containers and stored refrigerated and in darkness until fertilization. One control group was made with the diluted unirradiated milt. Each of the thirteen sperm aliquots were used to fertilize groups of 1000 salmon eggs which were left to hydrate in 0.5 L PE bottles at 8 °C until pressure treatment. At 3798 (3800), 4000 (4000), 4193 (4200), 4403 (4400), 4605 (4600) and 4816 (4800) minutedegrees (minC) (minutes*degree Celsius) the PE bottles were transferred to the pressure chamber and the eggs were subjected to a hydrostatic pressure of 655 bar (TRC-APV, Aqua Pressure Vessel, TRC Hydraulics inc., Dieppe, Canada) for 5 mins (the simplified group name is shown in brackets). Also, one batch of eggs fertilized with unirradiated milt were sampled every 10 mins from 540 mins (4320 minC) to determine the first cleavage interval (FCI). The samples (10–15 eggs) were cleared in 10% acetic acid before inspection.

On 12 December 2012 (Exp2012), 6 aliquots of diluted (1:40) sperm were made. Five of these were irradiated (0.45 mWcm^− 2^) and stored as described above. The last aliquot was left as an unirradiated control. Five egg groups of ~ 1500 (between 1239 and 1788) salmon eggs were fertilized with UV irradiated sperm (6 mins) and one group was fertilized with the control sperm. Hydration and pressure treatment was according to Exp2011, but treatments were done at 4713 (4700), 4800 (4800), 4899 (4900), 5011 (5000) and 5123 (5100) minC in addition to the control fertilization.

### Fish management and rearing

Eggs from Exp2011 were incubated at approximately 6 °C. From 23 April 2012 surviving larvae from the different groups were fed at 12 °C in individual square grey, covered, fibreglass tanks (1 × 1 × 0.25 m) and the temperature was switched to natural temperature in June 2012. All tanks were fed a commercial salmon feed in excess (Nutra Olympic, Skretting AS, Averøy, Norway) with automatic feeders (ARVO-TEC T Drum 2000, Arvotec, Huutokoski, Finland). Feed was given in small portions through the continuous light photoperiod. For illumination, two 18 W fluorescent daylight tubes (OSRAM L 18 W/840 LUMILUX, OSRAM GmbH, Ausburg, Germany) were used to produce 960 lx under water in the centre of the tank. Photoperiod and feeding were controlled automatically by a PC operated system (Normatic AS, Norfjordeid, Norway). On 7 August 2012 the surviving fish were PIT tagged and a small fin-clip was taken for genotyping. After tagging and sampling the experimental fish (including 45 control fish) were transferred to three tanks (1.5 × 1.5 × 0.7 m) for further ongrowing in common garden conditions.

Body mass and fork length were collected at eight time points; Aug 2012, Nov 2012, Jun 2013 (transfer to SW), Nov 2013, Jul 2014, Jun 2015 and Nov 2016. At each sampling time, fish were anaesthetized in 100 mgL^− 1^ Finquel® (MS 222). In Nov 2012, some stunted individuals were euthanized together with deformed fish (mainly curvatures and shortening of the vertebral column; see reduction in numbers in Additional file 1: Table S1; Fig. 1). Thirty-four control fish were also euthanized. In June 2013, 30 fish from the treatment groups and the remaining 11 controls were radiographed and checked for vertebral deformities. The controls were euthanized during the sampling. When the fish were measured in November 2013 and July 2014 fish were also euthanized (30 fish each time) to get samples for several other studies. Euthanasia was always done in 500 mgL^− 1^ Finquel® (MS 222) followed by exsanguination.

Fish were sexed by visual examination of the gonads at the time of terminal sampling. The condition factor (K) was calculated as K = 100 x weight (g) x length^− 3^ (cm). Specific growth rate (SGR, % per day) was calculated from the formula: SGR = (e^q^ − 1) × 100 [61], where q = [In(W_2_) − In(W_1_)] (t_2_-t_1_)^− 1^ [62] and where W_2_ and W_1_ were the live body weights at times t_2_ and t_1_, respectively. Levels of sexual maturation, based on external morphology, were assessed throughout.

Rearing of the fish produced in Exp2012 was the same as in Exp2011. Surviving larvae were first fed from 22 March. On 18 September 2013 the surviving progeny (240 fish from treatment groups and 30 controls) were tagged with passive integrated transponder (PIT) tags and tissue sampled. From Exp2012 only the survival data and genotyping are presented here.

### Production of clonal lines

In 2014, six females from Exp2011 were sexually mature. On 4 December 2014 they were all ovulated and their eggs were hand stripped and fertilized with three aliquots of diluted and UV irradiated sperm (same protocol as described above with 6 mins irradiation at 454 mW). Eggs were left to hydrate in 2 L PE bottles at 8 °C until pressure treatment. At 300 minC (second meiotic division [63]) the PE bottles containing eggs were transferred to the pressure chamber and they were thereafter pressurized for 5 mins at 655 bar. The weight of the females after stripping, the weight of the drained egg mass and the weight of 100 eggs, were recorded.

Rearing conditions for the fish in the clonal lines were the same as for the production of the double haploids described above. Survival to the eyed stage was registered on 10 February 2015 and surviving larvae were counted and first fed from 9 April 2015. On 8 June 2015 (day 60 of feeding), surviving fish were counted, distributed to other experiments and a small number were reared on for sampling and phenotypic description. On 16 November 2015, 8–10 fish from each clonal line were euthanized (500 mgL^− 1^ Finquel® (MS 222) and fin tissue samples were taken for microsatellite DNA analysis. On 30 November 2015, the remaining fish were euthanized, a blood sample taken for ploidy determination, length, weight, sex and deformities in the vertebral column were recorded. Deformities recorded included curvatures (lordosis, scoliosis and kyphosis) and/or shortening (short trunk and short tail [64]. Deformities in the head skeleton (upper and lower jaw and opercula) were recorded and as the pelvic fin was found to be missing in some individuals, this was also recorded.

### Genotyping

DNA was extracted from fin-clips. This was performed in 96-well plates using a commercially available extraction kit (Qiagen DNeasy®96 Blood & Tissue Kit). Each 96-well plate included two blank wells as negative controls. The samples were subject to genotyping with a set of 18 microsatellites that are routinely used in the molecular genetics laboratory at the Institute of Marine Research for Atlantic salmon genetics projects including ploidy determination (e.g. [65–67]). The samples taken from Exp2011 and 2012 were analyzed with all 18 of the microsatellites and the clonal fish produced in 2014 were analyzed with 16 of these. These loci were amplified in three multiplexes, using standard protocols for fresh tissues (Additional file 3); *SSsp3016* (Genbank no. AY372820), *SSsp2210*, *SSspG7*, *SSsp2201*, *SSsp1605*, *SSsp2216* [68], *Ssa197*, *Ssa171*, *Ssa202* [69], *SsaD157*, *SsaD486*, *SsaD144* [70], *Ssa289*, *Ssa14* [71], *SsaF43* [72], *SsaOsl85* [73], *MHC I* [74] and *MHC II* [75]. Polymerase chain reaction (PCR) products were analysed on an ABI 3730 Genetic Analyser and sized by a 500LIZ™ size-standard. The raw data was checked manually twice.

### Statistical analysis

Data were analysed in GraphPad Prism, version 6.0. Significance was assigned at *p* ≤ 0.05. Length, weight, and *K* factor data were first checked for normality within group using the Shapiro-Wilk test. Subsequently, parametric data were analysed using one-way ANOVA whereas non-parametric data were analysed using the Kruskal-Wallis test with treatment (3–6 levels depending on the timepoint) as a categorical variable. When main effects were significant, we used Tukey’s or Dunn’s multiple comparisons tests for parametric and non-parametric data, respectively. Each time point was analysed separately.

## Supplementary information

**Additional file 1 Table S1.** Mean ± SD (N) weight, length and condition factor *(*K*)* and sexual maturation (N mature of total N) of control fish and fish from the different treatment protocols.

**Additional file 2 Table S2.** Microsatellite analysis of 2 parents and 362 offspring from Exp2011 (row 1–366). The first 45 individuals (controls) were fertilized with diluted, but unirradiated milt. The others were fertilized with UV-irradiated milt and pressure shocked. Alleles that are exclusive for the Sire (sperm donor) are in green and blue and for the female in red and yellow. Only two individuals from the putative mitotic gynogenetic groups showed any paternal contribution (rows 55 and 216). The females that matured in 2014 and were used to produce the isogenic lines are shown in col. A with the number indicating which line they originated from. Rows 369 to 424 compares the microsatellites of the clonal founders with their progeny. Microsatellite data from Exp2012 is shown in lines 430 to 702 with the parents in lines 430 and 431, the doubled haploid progeny in lines 433 to 672 and the controls in lines 673 to 702.

**Additional file 3.** Details on condition of PCR reactions

## Data Availability

All relevant data and materials for this study are available from the first author Tom Hansen: tomh@hi.no.
