# Supplementary material for: Production and verification of the first Atlantic salmon (Salmo salar L.) clonal lines
Source: BMC Genet. 2020 Jul 8;21:71. doi: 10.1186/s12863-020-00878-8 (PMC7346428; doi:10.1186/s12863-020-00878-8)
Supplement: Supplementary file 1 — Additional file 1 Table S1. Mean ± SD (N) weight, length and condition factor (K) and sexual maturation (N mature of total N) of control fish and fish from the different treatment protocols. [file 12863_2020_878_MOESM1_ESM.docx]

Additional file 1: Table S1. Mean ± SD (N) weight, length and condition factor *(*K*)* and sexual maturation (N mature of total N) of control fish and fish from the different treatment protocols.

| **Parameter** |  | **Date** | **Control** | **Treatment groups (timing of pressure shock/min UV irradiation of sperm)** | | | | |
| --- | --- | --- | --- | --- | --- | --- | --- | --- |
|  |  |  |  | **4800/8** | **4800/6** | **4600/8** | **4600/6** | **4400/8** |
| Weight |  | Aug 2012 | 18 ± 3 (45) | 18 ± 5 (150) | 18 ± 5 (135) | 17 ± 6 (17) | 15 ± 7 (11) | 9 ± 8 (2) |
|  |  | Nov 2012 | 94 ± 14 (45) | 88 ± 28 (144) | 89 ± 27 (132) | 91 ± 25 (16) | 79 ± 32 (11) | 48 ± 40 (2) |
|  |  | Jun 2013 | 350 ± 72 (12) | 409 ± 115 (115) | 402 ± 117 (111) | 443 ± 99 (14) | 392 ± 111 (10) | 347 (1) |
|  |  | Nov 2013 |  | 1165 ± 284 (116) | 1157 ± 285 (111) | 1328 ± 218 (14) | 1134 ± 244 (10) | 1074 (1) |
|  |  | Jul 2014 |  | 3191 ± 757 (104) | 3048 ± 730 (96) | 3449 ± 569 (13) | 3134 ± 514 (9) | 3259 (1) |
|  |  | Jun 2015 |  | 7165 ± 1996 (87) | 6887 ± 1949 (82) | 7728 ± 1911 (9) | 6713 ± 1943 (8) |  |
|  |  | Nov 2016 |  | 10320 ± 2475 (8) | 9638 ± 2274 (11) | 13830 ± 1131 (2) |  |  |
| Length |  | Aug 2012 | 11.1 ± 0.5 (45) | 10.7 ± 1.1 (150) | 10.8 ± 1.2 (135) | 10.5 ± 1.6 (17) | 10.0 ± 1.5 (11) | 9.1 ± 2.2 (2) |
|  |  | Nov 2012 | 19.2 ± 0.9 (45)^a^ | 18.2 ± 2.2 (144)^b^ | 18.3 ± 2.1 (133)^b^ | 18.5 ± 1.9 (16)^ab^ | 17.4 ± 3.1 (11)^ab^ | 14.7 ± 4.2 (2) |
|  |  | Jun 2013 | 31.0 ± 2.2 (12) | 31.1 ± 3.2 (115) | 30.7 ± 3.3 (111) | 32.1 ± 2.3 (14) | 30.5 ± 3.1 (10) | 29.2 (1) |
|  |  | Nov 2013 |  | 43.3 ± 3.6 (116) | 42.8 ± 4.1 (111) | 45.4 ± 2.4 (14) | 42.3 ± 3.4 (10) | 42.0 (1) |
|  |  | Jul 2014 |  | 61.1 ± 4.6 (104) | 60.3 ± 4.8 (95) | 63.1 ± 3.2 (13) | 60.1 ± 3.7 (9) | 62.0 (1) |
|  |  | Jun 2015 |  | 78.0 ± 6.3 (87) | 77.1 ± 6.2 (82) | 80.2 ± 5.1 (9) | 75.9 ± 5.9 (8) |  |
|  |  | Nov 2016 |  | 91.1 ± 5.0 (8) | 90.4 ± 4.7 (11) | 97.0 ± 1.4 (2) |  |  |
| Condition |  | Aug 2012 | 1.33 ± 0.14 (45)^a^ | 1.38 ± 0.10 (150)^a^ | 1.42 ± 0.11 (135)^b^ | 1.41 ± 0.13 (17)^ab^ | 1.42 ±0.16 (11)^ab^ | 0.99 ± 0.39 (2) |
|  |  | Nov 2012 | 1.32 ± 0.06 (45)^a^ | 1.40 ± 0.11 (144)^b^ | 1.42 ± 0.14 (132)^b^ | 1.39 ± 0.11 (16)^ab^ | 1.38 ± 0.13 (11)^ab^ | 1.32 ± 0.10 (2) |
|  |  | Jun 2013 | 1.17 ± 0.07 (12)^a^ | 1.34 ± 0.24 (115)^b^ | 1.36 ± 0.24 (111)^b^ | 1.32 ± 0.09 (14)^b^ | 1.35 ± 0.09 (10)^b^ | 1.39 (1) |
|  |  | Nov 2013 |  | 1.41 ± 0.13 (116) | 1.46 ± 0.27 (111) | 1.41 ± 0.08 (14) | 1.48 ± 0.10 (10) | 1.45 (1) |
|  |  | Jul 2014 |  | 1.37 ± 0.10 (104) | 1.36 ± 0.13 (95) | 1.36 ± 0.08 (13) | 1.43 ± 0.08 (9) | 1.37 (1) |
|  |  | Jun 2015 |  | 1.47 ± 0.17 (87) | 1.46 ± 0.20 (82) | 1.47 ± 0.16 (9) | 1.49 ± 0.14 (8) |  |
|  |  | Nov 2016 |  | 1.35 ± 0.19 (8) | 1.29 ± 0.17 (11) | 1.52 ± 0.19 (2) |  |  |
| Sexual |  | 2014 |  | 6 of 104 | 0 of 95 | 0 of 13 | 0 of 9 | 0 of 1 |
| maturation |  | 2015 |  | 32 of 87 | 31 of 82 | 5 of 9 | 4 of 8 |  |
|  |  | 2016 |  | 6 of 8 | 7 of 11 | 2 of 2 |  |  |
