# Supplementary material for: Production and verification of the first Atlantic salmon (Salmo salar L.) clonal lines
Source: BMC Genet. 2020 Jul 8;21:71. doi: 10.1186/s12863-020-00878-8 (PMC7346428; doi:10.1186/s12863-020-00878-8)
Supplement: Supplementary file 3 — Additional file 3. Details on condition of PCR reactions [file 12863_2020_878_MOESM3_ESM.docx]

| **Promul 1** |  |  |  |
| --- | --- | --- | --- |
|  |  |  |  |
| **Component** | **Stock Concentration** | **Final Concentration** | **Final Volume (µl) 1 sample** |
|  |  |  |  |
| DNA | 16,50 ng/µl | 33,00 ng | 2,00 |
| Colorless GoTaq® Flexi Buffer | 5 x | 1 x | 2,00 |
| MgCl_2_ Solution | 25,00 mM | 2,00 mM | 0,80 |
| dNTP | 1,25 mM | 0,20 mM | 1,60 |
| GoTaq® G2 Flexi DNA polymerase | 5 u/µl | 0,35 u | 0,07 |
| SSsp2210-F/R* | 10,00 µM | 0,08 µM | 0,08 |
| SSspG7-F/R* | 10,00 µM | 0,20 µM | 0,20 |
| SsaD144-F/R* | 10,00 µM | 0,23 µM | 0,23 |
| Ssa202-F/R* | 10,00 µM | 0,08 µM | 0,08 |
| Sp2201-F/R* | 10,00 µM | 0,25 µM | 0,25 |
| SsaD157-F/R* | 10,00 µM | 0,25 µM | 0,25 |
| H_2_O |  |  | 2,44 |
| **Total** |  |  | **10,00** |
| *Forward and reverse primer is premixed. | |  |  |
|  |  |  |  |
|  |  |  |  |
| **Promul 2** |  |  |  |
|  |  |  |  |
| **Component** | **Stock Concentration** | **Final Concentration** | **Final Volume (µl) 1 sample** |
|  |  |  |  |
| DNA | 16,50 ng/µl | 33,000 ng | 2,000 |
| Colorless GoTaq® Flexi Buffer | 5 x | 1 x | 2,000 |
| MgCl_2_ Solution | 25,00 mM | 2,000 mM | 0,800 |
| dNTP | 1,25 mM | 0,200 mM | 1,600 |
| GoTaq® G2 Flexi DNA polymerase | 5 u/µl | 0,35 u | 0,070 |
| Ssa289 F/R* | 10,00 µM | 0,400 µM | 0,400 |
| Ssa14 F/R* | 10,00 µM | 0,120 µM | 0,120 |
| Sp1605 F/R* | 10,00 µM | 0,150 µM | 0,150 |
| Ssa171 F/R* | 10,00 µM | 0,150 µM | 0,150 |
| Sp2216 F/R* | 10,00 µM | 0,015 µM | 0,015 |
| H_2_O |  |  | 2,695 |
| **Total** |  |  | **10,000** |
| *Forward and reverse primer is premixed. | |  |  |
|  |  |  |  |
|  |  |  |  |
| **Promul 3** |  |  |  |
|  |  |  |  |
| **Component** | **Stock Concentration** | **Final Concentration** | **Final Volume (µl) 1 sample** |
|  |  |  |  |
| DNA | 16,50 ng/µl | 33,00 ng | 2,00 |
| Colorless GoTaq® Flexi Buffer | 5 x | 1 x | 2,00 |
| MgCl_2_ Solution | 25,00 mM | 2,00 mM | 0,80 |
| dNTP | 1,25 mM | 0,20 mM | 1,60 |
| GoTaq® G2 Flexi DNA polymerase | 5 u/µl | 0,35 u | 0,07 |
| SsaF43 F/R* | 10,00 µM | 0,05 µM | 0,05 |
| Ssa197 F/R* | 10,00 µM | 0,05 µM | 0,05 |
| SsaD486 F/R* | 10,00 µM | 0,04 µM | 0,04 |
| SSsp3016 F/R* | 10,00 µM | 0,05 µM | 0,05 |
| MHC1 F/R* | 10,00 µM | 0,05 µM | 0,05 |
| MHC2 F/R* | 10,00 µM | 0,08 µM | 0,08 |
| SsOSL85 F/R* | 10,00 µM | 0,18 µM | 0,18 |
| H_2_O |  |  | 3,03 |
| **Total** |  |  | **10,00** |
| *Forward and reverse primer is premixed. | |  |  |
|  |  |  |  |
| **Thermal cycling conditions Promul1:** | |  |  |
|  |  |  |  |
| **Step** | **Temperature (°C)** | **Time** | **Number of cycles** |
| Denaturation | 94 | 4 minutes | 1 |
| Denaturation | 94 | 50 seconds | 31 |
| Annealing | 55 | 50 seconds |  |
| Extension | 72 | 80 seconds |  |
| Final extension | 72 | 10 minutes | 1 |
| Soak | 4 | Indefinite | 1 |
|  |  |  |  |
| **Thermal cycling conditions Promul2 and 3:** | |  |  |
|  |  |  |  |
| **Step** | **Temperature (°C)** | **Time** | **Number of cycles** |
| Denaturation | 94 | 4 minutes | 1 |
| Denaturation | 94 | 50 seconds | 30 |
| Annealing | 55 | 50 seconds |  |
| Extension | 72 | 80 seconds |  |
| Final extension | 72 | 10 minutes | 1 |
| Soak | 4 | Indefinite | 1 |
